# Annonaceae: Breaking the Wall of Inflammation

**DOI:** 10.3389/fphar.2017.00752

**Published:** 2017-10-20

**Authors:** Ali Attiq, Juriyati Jalil, Khairana Husain

**Affiliations:** Drug and Herbal Research Centre, Faculty of Pharmacy, Universiti Kebangsaan Malaysia, Kuala Lumpur, Malaysia

**Keywords:** anti-inflammatory, annonaceae, cytokines, prostaglandins, nuclear factor kappaB, reactive oxygen species, cycloxegenase

## Abstract

Inventories of tropical forests have listed Annonaceae as one of the most diverse plant families. For centuries, it is employed in traditional medicines to cure various pathological conditions including snakebite, analgesic, astringent, diarrhea, dysentery, arthritis pain, rheumatism, neuralgia, and weight loss etc. Phytochemical analysis of Annonaceae family have reported the occurrence of alkaloids, flavonoids, triterpenes, diterpenes and diterpene flavone glycosides, sterols, lignans, and annonaceous acetogenin characteristically affiliated with Annonaceae sp. Numerous past studies have underlined the pleotropic pharmacological activities of the crude extracts and isolated compounds from Annonaceae species. This review is an effort to abridge the ethnobotany, morphology, phytochemistry, toxicity, and particularly focusing on the anti-inflammatory activity of the Annonaceae species.

## Introduction

Inflammation is a human body's defense mechanism that can be triggered by numerous factors including physical trauma, exposure to allergen, chemical or heat stimulus, and microbial infection (Kidd and Urban, 2001; Guo et al., 2015) The inflammation is a tightly regulated process involving pro-inflammatory stimulus, to initiate and maintain inflammation and anti-inflammatory signals which helps in shutting down the process (Dinarello, 1997; Ziebell and Morganti-Kossmann, 2010). However, there are several internal or external factors that can disturb this regulatory network of pro and anti-inflammatory mediators (Cytokines, chemokine etc.), and hence giving rise to various inflammatory conditions including inflammatory bowel disease rheumatoid arthritis, multiple sclerosis, and chronic asthma (Sahlmann and Ströbel, 2016). Several therapeutic options like non-steroidal inflammatory drugs (NSAIDs), corticosteroids and Disease Modifying Anti Rheumatic Drugs (DMARDs) are now available to treat these life threatening and painful inflammatory conditions (Hoes et al., 2010; Weber and Noels, 2011). However, prolong treatment with these drugs have been associated with serious and sometimes life threatening side effects including uncontrolled hypertension, gastric ulcers, acute kidney failure, glaucoma, heart failure etc., (Jachak, 2006; Huscher et al., 2009).

Natural products have been the counter stone for the traditional medicine practices around the world for centuries. Throughout this time numerous plant species have been used in the form of tinctures, decoctions, and dried powder form to treat all sort of inflammatory condition (Van Wyk and Wink, 2004). The knowledge of medicinal activity has been gathered over the course of centuries, using personal observations and trial and error methods. Hence it is imperative to carry out further research on plants species with medicinal properties to validate their therapeutic activity. During the last century, natural products have proved to be an essential pipeline for drug discovery and drug design (Al-Dhubiab, 2012) and had gain great significance due to their extraordinary value in the field of pharmacology and therapeutics (Kumar and Khanum, 2012; Moghadamtousi et al., 2013; Akaberi et al., 2015).

Annonaceae has been listed among the most diversified families of tropical forest due to its heterogeneity and abundance in this region (Phillips and Miller, 2002; Couvreur et al., 2011). This review is an effort to highlight the botanical features, phytochemistry, medicinal uses, and anti-inflammatory activities and toxicity of most notable species of Annonaceae. Moreover, this review has analyzed the scientific data from experimental studies to validate the traditional uses and claimed anti-inflammatory activities of few Annonaceae species.

## Classification and botanical features of annonaceae species

The botanical features of the Annonaceae family can vary from species to species depending upon its origin, climate, and topography. Its botanical diversity can range from trees to shrubs, evergreen climbers, with elongated cylindrical-shaped intracellular resin channels and broad and well-developed septate pith in the stems (Hamonnière et al., 1977). The aromatic flowers bloom before they are completely developed; they are axillary, singular or grouped, hermaphrodite, and regular in shape. The stamens are typically considerable in numbers, hypogenous, and spirally arranged. Fruits are made up of clusters of berries and that are widely consumed in tropical regions due to their high nutritional value. Seeds are usually enlarged and have irregular surfaced endosperm with a small embryo (Hutchinson, 1959; Hamonnière et al., 1977; Takhtajan, 2009).

For over a considerable period of time botanists have confused and mistakenly included monotypic Eupomatiaceae in Annonaceae family, but the differential botanical features of Annonaceae have made it easier to differentiate Annonaceae from Eupomatiaceae. For instance the presence of vessel perforations of leaves, broad and high multi seriate xylem rays which give a cross-sectional “cobweb-like” wood structure are characteristically associated with Annonaceae species (Koek-Noorman and Westra, 2012). Moreover, the distichous phyllotaxis leaves, petals, sepals, and stamens are in the sets of three (trimerous) (Watson and Dallwitz, 1999). While calyx and corolla, long chalaza are in a perfect symmetrical ovule (Sauquet and Le Thomas, 2003; Sauquet et al., 2003).

The first classification of Annonaceae family was carried out by Dunal (1817). The classification of Dunal was solely based on fruit morphology. Later, Baillon (1868) and Diels and Alder (1932) used flower characters to develop a new classification of Annonaceae family. However, the classification of Fries (1959) was comprehensive and most authentic. He improvised the pervious classifications by combining floral characteristics and fruit morphology and hence these interventions made the classification of Fries to be a gold stranded for future taxonomical studies as well (Zomlefer, 1994).

The presence of many primitive and archaic morphological features and their ability to survive the mass extinctions has characterized the Annonaceae species as “living fossils.” The order Magnoliales is among the largest orders, comprising six families including Eupomatiaceae, Myristicaceae, Magnoliaceae, Degeneriaceae, Himantandraceae, and Annonaceae (Davis and Wurdack, 2004; Soltis and Soltis, 2004). With 180 genra with more than three thousand species, Annonaceae family is the largest family of this order. Annonaceae species are predominantly distributed in parts of world with abundant rainfall including Malaysia, Indonesia, Japan, India, Sri Lanka, and Pakistan.

## Traditional uses

Natural products have been the basis of many traditional medicines from all around the world. Moreover, these medicinal practices have played a significant role in the providing the remedies for all sorts of inflammatory conditions. Annonaceae family is very famous in tropical regions due to its widespread use in traditional medicine For instance, the juice of the macerated leave of *Annona muricata* is used in Brazil for arthritis, rheumatism, and neuralgia (Cercato et al., 2015). Moreover, in some parts of Indonesia the dried leave are orally ingested for its potent analgesic effect (Badrie and Schauss, 2010; Bele et al., 2011). Moreover, leaves of *Anaxagorea dolichocarpus*, commonly known Jari Jari has been a traditional remedy of articular rheumatisms for centuries. Native South African tribes tropically apply the gridded Jai Jari leaves with castor oil to treat articular rheumatisms (DeFilipps et al., 2004). In Brazil, fresh fruit of *Annona dioica* is used due to its long history of wound healing in fresh wounds (Formagio et al., 2013a). Likewise in Amazona, the northwestern part of Brazil *Duguetia chrysocarpa* leaves and twigs are ground together and extract of this mixture is used as a remedy for inflammatory bowl diseases and gastrointestinal ulcers (Almeida et al., 2011, 2012). While, in other parts of the world have benefited from the seed, leave and fruits of *Annona reticulata*. Counties like West Indies and Dominican Republic use fruit decoction as a traditional remedy for bronchitis. While, oral ingestion of the powdered leaves are reported to decease the frequency and intensity of asthma attack (Auddy et al., 2003; Bhalke and Chavan, 2011). Peninsular Malaysian rain forests are famous for their ecological diversity and due to this heterogeneity in plant species it has been origin of numerous household remedies for inflammatory diseases. For instance, *Cananga odorata* commonly known as perfume tree is very famous in this region due to its strong anti-pyretic and anti-inflammatory activity (Duke and Beckstrom-Sternberg, 2000). Fresh wounds are wash with bark extract and decoction to reduce inflammation and facilitates the wound healing (Tan et al., 2015). While, the extract is poured into the eye in order to reduce the ophthalmic inflammation (Scartezzini and Speroni, 2000; Rahman et al., 2005). Likewise, in Sabah and Sarawak, western part of the Malaysia several Annonacaeae species including *Enicosanthellum pulchrum, Friesodielsia latifolia, Uvaria grandi, Uvaria rufa* are in used a remedy for pedal edema (Nordin et al., 2014), generalized body pain (Araujo et al., 2017), antipyretic (Parmar et al., 1994), and anti-inflammatory (Buncharoen et al., 2016). Moreover, in Africa 80% of the population is still dependent on the traditional remedies due to easy accessibility and low cost. Several indigenous species including *Enantia* chlorantha have been reported to possess strong immunomodulatory activity. The Powdered bark is mixed with citrus lemon and then paste is used as dressing for artharitis (Tan et al., 2000). While, in Sudan ethanolic root extract and whole fruit of *Xylopia aethiopica* is used as a remedy for neuralgia, headache and colic pain (Ogunkunle and Ladejobi, 2006; Woode et al., 2012). While in central Africa the root decoction of *Xylopia parvifolia* is as a natural remedy for pain management and roots are chews and then swallowed for gastrointestinal ulcers and inflammations. The traditional uses of most widely used Annonaceae species are summarized in Table 1 along with the parts used and mode of administration.

**Table 1:** Medicinal uses of most commonly used Annonaceae species.

**Plant name**	**Country region**	**Local name/Common name**	**Medicinal uses**	**Part (used)**	**Mode of usage/preparation**	**References**
*Alphonsea javanica* Scheff.	Indonesia	Aku Battu	Rheumatism and edema	Leave	Ethanolic extract	Johnson et al., 2013
*Annona crassiflora* Mart.	Cerrado biome	Araticum of the Cerrado, marolo, or panã	Rheumatism, wounds healing, healing, and anti-inflammatory	Fruit	No information	Vilar et al., 2008; Silva et al., 2014
*Annona dioica* St. Hill	Brazil	Ceraticum and ariticum	Rheumatism	Fruits and leaves	Dried leave paste and fresh fruit decoction	Formagio et al., 2013a
*Annona muricata* Linn	Brazil	Araticum, condessa, graviola	Analgesic, Arthritis pain, rheumatism, neuralgia	Fruit Leaf	Juice of fresh fruit and water extract of leaves (12 h of cold maceration)	Badrie and Schauss, 2010; Cercato et al., 2015
*Annona reticulata* Linn	West Indies	Ramphal	Bronchitis, Asthma, Bowel inflammation	fruit Seeds Leaf	Oral ingestion of the leaf powder Decoction of fruit in boiled water. Powdered seeds ingested	Auddy et al., 2003; Bhalke and Chavan, 2011
*Annona senegalensis Persoon*	Nigeria	Ukopko (Idoma)	Anti-inflammatory and analgesic	Leaf Root	Roots and bark are ground together and their Decoction is used	Ofukwu et al., 2008
*Annona vepretorum* Mart	Brazil	Araticum, bruteira	Analgesic and anti-inflammatory	Leave	Methanolic Leaf extract	Silva et al., 2015
*Cananga odorata* Hook.f. and Thomson	Malaysia and India	Kenanga utan, perfume tree, sananga oil, kenanga wood	Rheumatism Ophthalmic inflammation and Wound healing	Bark	Decoction is used to wash fresh wounds and extract dropped into eyes for inflammation	Duke, 2000; Scartezzini and Speroni, 2000; Rahman et al., 2005
*Duguetia chrysocarpa* Maas	Brazil	Pindaíba-da-mata	Rheumatism and Bowl inflammation	Leave and twigs	Powdered leaves and twigs extract is drunk to relive inflammatory conditions	Almeida et al., 2011, 2012
*Enantia chlorantha* var. soyauxii Engler and Diels	Africa	African yellow wood	Arthritis and wound healing	Bark	Powdered bark with citrus lemon used as dressing	Tan et al., 2000
*Enicosanthellum pulchrum* King) Heusden	Malaysia	Disepalum	Rheumatism fever, asthma, and edema	Leave	Fine powder can ingested directly or water decocotion can also be used for asthma and rheumatism	Nordin et al., 2014
*Fissistigma oldhamii* (Hemsl.) Merr	Southern China	Oldhamii	Rheumatoid arthritis	Stems and roots	Dried stem and root powder is orally ingested	Araujo et al., 2017
*Friesodielsia latifolia* Hook.f. and Thomson	Malaysia	No information	Gernalized body pain and Pedal edema	Roots	Root decoction	Wiart, 2007
*Mitrella Kentii* (Blume) Miq	Indonesia	Kiawi	Antipyretics and Edema	Roots	Decoction to treat fever	Wiart, 2006
*Monodora myristica* (Gaertn.) Dunal	Ivory coast	*M Kpo*. Abidjan district	Febrile pains, eye diseases and hemorrhoids and headaches	Fruits Seed	Seeds and fruits consumed in whole or ground to be used in soup and strewed	Moukette et al., 2015
*Polyalthia longifolia* cv. Pendula	India	Ashoka	Fever	Bark	Bark decoction	Chadha, 1985; Katkar et al., 2010
*Uvaria grandiflora* Roxb. ex Hornem	Malaysia	*Pisang Tandok*	Wound healing, Fever with chills and rigors	Leaves	Paste of the leaves wrapped around the abdomen part of children	Parmar et al., 1994
*Uvaria rufa* Blume	Malaysia	Larak or Pisang-pisang	Antipyretic and anti-inflammatory	Leave Bark	Soaked leaves in water 50% ethanolic extract of bark	Buncharoen et al., 2016
*Xylopia aethiopica* (Dunal) A.Ric	Sudan	Ethiopia or Negro pepper	Rheumatism, headache, colic pain, and neuralgia	Fruits Seeds	Ethanolic fruit extract and The dried fruits are used as whole	Ogunkunle and Ladejobi, 2006; Woode et al., 2012
*Xylopia aromatic* Lam. Mart	Columbia an brazil	Monkey pepper	Pulmonary inflammation and hemorrhoids	Roots Leaves	Insertion of root pieces into rectum and leaves burnt and smoke inhaled	Woguem et al., 2014
*Xylopia parvifolia* Hook.f. and Thomson	East and Central Africa, India	Netawu/Athu ketiya	Gastrointestinal ulcers and analgesic	Roots	Decoction Finely drinded powder	Nishiyama et al., 2006

## Anti-inflammatory activity

Portfolios of tropical forests perpetually list Annonaceae as one of the most diverse plant families (Phillips and Miller, 2002). With respect to the species abundance it contributes notably to the diversity of trees in Neotropical region (Saunders, 2012). Similarly, this diversity can also be seen in its wide range of phyto-constituents. Phytochemical studies of this family have reported the presence of alkaloid (Malebo et al., 2013; Kouam et al., 2014; Soares et al., 2015), cyclopeptides (Wu P. et al., 2014; Moghadamtousi et al., 2015), flavonoids (Lage et al., 2014; Chokchaisiri et al., 2015), terpenoids (Annan et al., 2013; Rabelo et al., 2016), and lignans (Moreira et al., 2013; Nguyen et al., 2015; Rayanil et al., 2016). Several bioactivities including antimalarial (Frausin et al., 2014; Meira et al., 2015), antiplatelet, (Thang et al., 2013a; González-Esquinca et al., 2014), and anti-inflammatory activity (Kandimalla et al., 2016) are just to name a few. Cytotoxicity of Annonaceous acetogenins has made this family of major interest for novel anti-cancer drugs (Han et al., 2015; Yang et al., 2015). However, the focus of this study is to demonstrate vast array potential compounds, for future drug discovery with anti-inflammatory activity. Annonaceae species have grave importance with relevance to its wide range of anti-inflammatory activities. *In vitro* and *in vivo* studies including isolated compounds as well as rudimentary crude extracts have shown potent activity in all sorts of inflammation. An in depth detail of Annonaceae species has been summarize in Table 2.

**Table 2:** Mechanism of action of extracts and isolates of Annonacae species with potent anti-inflammatory activity.

**Plant name**	**Part used**	**Isolate compound/Extract**	**Class**	**Mechanism of action/Conclusion**	**References**
*Alphonsea javanica* Scheff	Leaf	(+)-Altholactone and (+)-goniothalmin	Styryl-lactone	LPS induced NO production, IKB-α, and expression of iNOS and COX-2 was significantly reduced in Raw macrophage 264.7 cells at IC_50_ range of 0.8–5.0 μM	Johnson et al., 2013
*Annona crassiflora* Mart	Leave	Methanolic extract	–	Oral treatment with 100 and 300 mg/kg reduced carrageenan-induced edeme by 53 ± 7 and 47 ± 10% and leukocyte migration was suppressed by 60 ± 7 and 63 ± 7%, respectively	Rocha et al., 2016
*Annona Cheromola Mill*	Fruit	Ethanol, methanol and dimethyl formammide	–	Methanolic extracts has shown maximum Extract has exhibited potent radical scavenging activity toward 1,1-Diphenyl-2-picryl-hydrazyl and Superoxide anion at IC_50_ range of 100–250 μg/mL	Barreca et al., 2011
*Annona dioica* A.St.-Hil	Leaves	Quercetin and kaempferol	Flavonoids	Leukocytes migration activity was inhibited at IC_50_ Value of 8.53 and 10.57 μg /mL, respectively	Formagio et al., 2013a,b
*Annona glabra* L	Fruit	Isodesacetyluvaricin	Acetogenins	Selectively inhibited COX-2 and mRNA expression at dose 5 μM	Wu et al., 2012
	Fruits	7β,17-dihydroxy-ent-kaur-15-en-19-oic acid 19-O-β-d-glucopyranoside ester	Ent-kaurane diterpenoids	Significant inhibition in iNOS production was observed with an IC_50_ of 0.01 μM	Nhiem et al., 2015
*Annona montana* Macfed	Seeds	Cyclomontanins	Cyclopeptides	Significant inhibition in TNF-α and IL-6 production was observed in Murine macrophage J774A with an IC50 value of 30 μg/mL	Chuang et al., 2008
*Annona muricata* L	Leaves	Ethanolic extract	–	Reduced the number of abdominal contortions by 14.42% at 200 mg/Kg, increased the reaction time on a hot plate at doses of 200 mg/kg and carrageenan induced paw edema was reduced by 29.33% at 200 mg/kg	de Sousa et al., 2010
	Unripe fruit	Lyophilized fruit extract	–	Infiltrations of inflammatory meditators were significantly inhibited with pretreatment of 100 mg/mL extract in mice	Ishola et al., 2014
				Reduction in ROS and PGE_2_.production was observed at dose of 200 and 400 mg/kg in mice, respectively	Moghadamtousi et al., 2014b
	Leaf	Ethanolic extract	–	Significantly decreased of TNFα and IL-1β levels were reported in Freund's adjuvant induced arthritis mice at dose of 100 mg/kg	Foong and Hamid, 2012
	Leave	Aqueous Extract	–	Showed a significant decrease in elevated NO level in streptozotocin induced pancreatic β cells at 100 mg/kg. .	Adewole and Caxton-Martins, 2006
*Annona purpurea* Moc. and Sessé ex Dunal	Leaves	7-hydroxy-dehydrothalicsimidine, thalicsimidine, N-methyllaurotetanine, lirinidine, N-methylasimilobine	Alkaloids	PAF-induced platelet aggregation was inhibited at a concentration range of 20–50 μM	Chang et al., 1998
*Annona reticulata* L	Bark	Kaur-16-en-19-oic acid	Ent-kaurane Diterpenoid	Hot plate reaction time was increased, reduction in acetic acid-induced abdominal writhing and carrageenan induced rat paw edema was observed at 20 mg/kg	Chavan et al., 2012
	Bark	Methanolic extract	–	Pretreatment with 200 μg/mL have shown significantly neuro-protective response by inhibition of NF-κB inflammatory cascade leading to suppression of IL-1β, IL-6, IL-10, TNF-α, and iNOS in SHSY5Y cells and DRG neuronal cells	Kandimalla et al., 2017
	Leaves	Kaurenoic acid, taraxerol, 16α-hydro-19-al-ent-kauran-17-oic acid, 6β-hydroxystigmast-4-en-3-one, and 17-acetoxy-16β-ent-kauran-19-oic acid 24, 16α-hydro-ent-kauran-17,19-dioic acid	ent-kaurane diterpenoids	Significant NO and superoxide anion generation inhibitory activity was observed at IC_50_ value ranging from 5.25 to 8.65 μM	Thang et al., 2013b
*Annona senegalensis Pers*	Leaves	Ethanolic extract	–	Deceased the production of neutrophils, eosinophil and macrophages at 7 mg/kg	Yeo et al., 2011
*Annona squamosa L*	Bark	Caryophyllene oxide	Sesquiterpentine	Late phase of paw licking edema was significantly reduced at the dose of 50 mg/kg	Chavan et al., 2010
	Seeds	Cyclosquamosin and met-cherimolacyclopeptide	Cyclopeptides	IL-6 and TNF-α production was suppressed in J774A with an IC_50_ value of 1.22 and 9.2 μM	Dellai et al., 2010
	Fruit	Fanlizhicyclopeptide a fanlizhicyclopeptide	Cyclopeptides	Pro-inflammatory cytokine production was inhibited by 32 and 27%, TNF-α by 51 and 57 %, and IL-6 by 66 and 49% at 25 μM	Wu P. et al., 2014
	Stem	16beta,17-dihydroxy-ent-kauran-19-oic acid	Ent-kaurane	Suppressed the degranulation of neutrophils were suppressed through immobilization of cytosolic calcium in a concentration dependent manner at IC_50_ value of 12.52 μM	Yeh et al., 2005
	Leaves	–	–	Significant inhibition of NO (73.64%), moderate reduction in superoxide (89.77%), and lipid peroxidation (99.02%) was observed due to its potent scavenging activity at 1,000 μg/ml	Shirwaikar et al., 2004
*Annona sylvatica* A.St.-Hil	Leaves	Hinesol, z-caryophyllene, beta-maaliene,	Sesquiterpenes	Leukocytes migration was inhibited at a concentration range of 36.04–45.37 μg/mL	Formagio et al., 2013a,bc
*Annona vepretorum*	Leaves	crude ethanolic extract	–	Oral dose of 25, 50, 100 mg/kg inhibited the release of inflammatory mediators and leukocyte migration is inhibited by 59, 65, and 79%, respectively	Silva et al., 2015
*Cyathostemma Argenteum* (Blume) J.Sinclair		4′, 6′-dihydroxy-2′, 4-dimethoxy-5′-(2″-hydroxybenzyl) and dihydrochalcone dihydrochalcone,4′, 6′-dihydroxy-2′, 4- dimethoxydihydrochalcone	Chalcone	Release of inflammatory mediators and leukocyte migration was significantly inhibited in rats at concentration of 1 mg/ear	Somsrisa et al., 2013
*Duguetia chrysocarpa* Mass	Fruit	Discretamine	Alkaloid	Acetic acid-induced writhing, formalin and hot plate tests has shown significant results	Almeida et al., 2012
*Enicosanthellum pulchrum* (King) Heusden	Roots	Ethyle acetate extract	–	Produces platelet activating factor antagonistic activity at 85.6% inhibition at 250 mg/kg oral dose	Nordin et al., 2012
*Fissistigma cavaleriei* (H.Lév.) Rehder	Root	Compound 1 (name not illustrated by author)	Alkaloid	Suppress COX2 expression at IC_50_ value of 32 μg/mL	Yang Z. et al., 2012
*Fissistigma Oldhamii* (Hemsl.) Merr	Stem	Crude ethanolic extracts	–	TNF-α and IL-6 production and released suppressed at 50 μg/mL	Ge et al., 2013
	Stem	Isopedicin	Flavonoid	The production of ROS in neutrophils were inhibited due to the elevation of cellular cAMP and activation of protein kinase A through its inhibition of CAMP-specific phosphodiesterase at an IC_50_ value of 0.34 μM	Hwang et al., 2009
	Leaves	7′-(3′, 4′-dihydroxyphenyl)-n-[(4-methoxyphenyl) ethyl] propenamide (z23)	_	Z23 has decreased the gene expression of COX2 and iNOS at a concentration range of 6.25–20 μM	Hu et al., 2008
*Goniothalamus. clemensii*, Ban *Goniothalamus. Woodii* Mex ex Mat- Salleh, *Goniothalamus. velutinus* Mex ex Mat- Salleh *and Goniothalamus. tapis* Miq	Bark	Bark oil	Sesquiterpene and Sesquiterpenoid	PAF and arachidonic acid activity was significantly inhibited at 20 μg/mL	Vendramini-Costa et al., 2014
	Bark and root	Goniothalamin	Styryl-lactones	Goniothalamin has shown gastro-protective effect against ethanol-induced gastric ulcers due to its COX 1 stimulatory and Glutathione induction property at concentration range of 18–25 μg/mL	Vendramini-Costa et al., 2014
	Bark	Goniothalamin	Styryl-lactones	Gene expression and production of IL-6, IL-17, and TNF-α was down regulated and suppressed at a concentration range of 10-50 μg/mL	Vendramini-Costa et al., 2017
*Goniothalamus uvaroides* King *and Goniothalamus tapis* Miq	Bark	(+)-Goniothalamin and (+)-isoaltholactone	Styryl-lactones and sesquiterpene lactone	Significant PAF receptor antagonist activity was significantly was observed with an IC_50_ value of 19.7, 46.5 μM	Moharam et al., 2012
*Goniothalamus. clemensii*, Ban *Goniothalamus. Woodii* Mex ex Mat- Salleh, *Goniothalamus. velutinus* Mex ex Mat- Salleh *and Goniothalamus. tapis* Miq	Bark and Root	Ethyl Acetate Extract	Essential oils	PAF receptor antagonist activity and PAF induced platelet aggregation was significantly inhibited with an IC_50_ value of 93.3 and 87.7 μg/ml	Moharam et al., 2010a
*Guatteria australis* A.St.-Hil	Leaves	Ethyl acetate extract	Essential oils	Slight anti-oxidant activity was observed at 250 μg/ml.	Siqueira et al., 2015
*Goniothalamus macrophyllus* Bloom Hook.f. and Thomas	Leave and bark	(r)-(+)-goniothalamin (GTN)	Styryl-lactone	GTN inhibited TNF α induced NF-κb activation with an IC_50_ value of 5 μM	Orlikova et al., 2013
*Meiocarpidium Lepidotum Lepidotum* (Oliv) Engl. and Diels	Bark	Crude aqueous extract containing terpenoids	Triterpines	Significantly reduced the writhing, carrageenan–induced hyperalgesia in mice at concentration of 1mg/kg	Meddah et al., 2013
*Melodorum fruticosum* Lour	Leaves	Melodamide	Phenolic amide	Superoxide anion generation and elastase inhibition of neutrophils at an IC_50_ value ranging from 5.25 to 8.65 μM	Chan et al., 2013
*Mitrella kentia* (Blume) Miq	Leaves	Acetylmelodorinol, chrysin and polycarpol, benzoquinone and stigmasterol	Alkaloid	Significant and concentration dependent inhibition of PAF, PGE_2_ and thromboxane B2 at an IC_50_ value of 15.6, 19.1, 19.4 μM	Saadawi et al., 2012
	Leaves	Desmosdumotin	Chalcone	Selectively inhibited COX-2 by 29.5% and 34.8% at 250 and 500 ng/ml	Sidahmed et al., 2013
*Miliusa balansae* Finet and Gapnep	Leaves	Milbasides A, B and C	MegastigmaneGlycosides	LPS induced NO production was significantly reduced at a concentration range of 20–40 μM	Thao et al., 2015
*Monodora myristica* (Geartn.)Dunal	Seed	Hydro-ethanolic Extract	–	Inhibition of carrageenan-induced paw edema, and xylene-induced ear edema was significantly reduced at a concentration range of 50–200 mg/kg	Ishola et al., 2016
*Monodora tenuifolia* Benth	Seed	Methanolic crude extract	–	Concentration range of 400–800 μg/ml exhibited maximum effect against lipid peroxidation and free radical generation, exhibited significant antioxidant activity in NO induced lipid peroxidation	Njoku, 2007
*Oxandra xylopioides* Diels	Leaves	Berenbjenol	Cycloartane triterpene	Significantly reduced the IL-1 production by 72 and 81% and carrageenan induced paw edema by 64 nd 43 & at concentration of 100 μM	Rojano et al., 2007
	Bark	Berenjenol (berenjenol acetate and 3-oxo-berenjenol)	Cycloartane triterpene	Expression of COX-2 and iNOS was reduced by 65 and 80% at 50 μM	Aquila et al., 2009
*Polyalthia longifolia* (Sonn.) Thwaites	Bark	16-hydroxycleroda-13-ene-15,16-olide-3-one	Clerodane diterpenoid	Superoxide Anion generation was inhibited with an IC_50_ value of 0.60 ± 0.09 μg/ml	Chang et al., 2006
	Leaves	Pl3s [6-hydroxycleroda-3,13(14)e-dien- 15-oic acid]	Clerodane diterpenoid	Neutrophil respiratory burst and superoxide anion generation was significantly inhibited at 3.06 ± 0.20 and 3.30 ± 0.48 μM, respectively	Chang et al., 2008; Tanna et al., 2009
	Unripe fruit	16-hydroxycleroda-3,13-dien-15,16-olide (6) and 16-oxocleroda-3,13-dien-15-oic acid (7)	Diterpenes	At 10 μM iNOS production was significantly inhibited by 81.1 and 86.3% with an IC_50_ value of 1 μM	Wu T. H. et al., 2014
	Leaf	Ethanolic extract	–	Maximum NO scavenging activity was 70.67% with an IC_50_ value of 167 μg/ml	Saha et al., 2008
	Leaves	6-hydroxycleroda-3,13-dien-15,16-olide (pl3)	Diterpenes	Pre-treatment with 10 μg/ml pl3 notably decreased the production of NO, PGE_2_, iROS, and TNF α. Moreover, gene expression of NF-κB p65, COX-2, and iNOS was also suppressed	Shih et al., 2010
*Polyalthia parviflora* Ridl.	Leaves	13 6s-styryllactones, 6s-styrylpyrones and1s-phenylpyranopyrones	Styryllactones	Superoxide anion generation and elastase release from human neutrophils was suppressed were inhibited with an IC_50_ value of 30.1 ± 2.5 and 21.2 ± 2.2 μM, respectively	Liou et al., 2014
*Pseuduvaria macrophylla* (Oliv.) Merr	Bark	Crude methanolic extract	–	Diabetic rats treated with 400 mg/kg significantly inhibited the production of pro-inflammatory cytokines including TNF-α, IL-1β, IL-6	Arya et al., 2014
*Pseuduvaria monticola* J.Sinclair	Bark	Crude ethanolic extract	–	Daily administration of 500 mg/kg for 45 days down regulated the levels of oxidative stress and pro-inflammatory cytokines by inhibiting the translocation of NF-κB in type 2 diabetic rat model	Taha et al., 2014
*Polyalthia cerasoides* (Roxb.) Bedd	Leave	Methenolic leave extract	–	Hydroxyl radical, superoxide anion scavenging, and potent reducing activity was observed in rats treated with 40 mg/kg of extract	Ravikumar et al., 2008
*Rollinia mucosa* (Jacq) Baill	Leave and seeds	Magnolin, epiyangambin, yangambin	Lignans	Significant PAF receptor antagonist activity was recorded at a IC_50_ range of 1.1–6.7 μM	Faria Lua Figueiredo et al., 1999
	Stems	Romucosine A and D	Alkaloid	At 100 μg/ml maximum PAF receptor antagonist activity was recorded	Kuo et al., 2001
*Toussaintia orientalis* Verdc	Stem, root and bark	Aristolactam aii, aristolactam bii	Aristolactam alkaloid toussa lactam	Maximum inhibition of histamine release from mast cells via stabilizing the cell membrane was observed at IC_50_ value of 5.1 and 11.9 μM	Odalo et al., 2010
*Uvaria chamae* P.Beauv	Whole plant	Methanolic crude extract	–	Pretreatment with 400 mg/kg for 6 h inhibited paw circumference in the carrageenan- and formaldehyde-induced in rat paw oedema tests	Popoola et al., 2016
*Uvaria flexuosa* Ast & Jovet	Leaves	Flexuvarol b and chrysin	Flavones	Superoxide anion generation and elastase release from human neutrophils was suppressed at an IC_50_ value of 2.25–5.55 μM	Hsu et al., 2016
*Uvaria grandiflora* Roxb. Ex Hornem	Stem	(–)-Zeylenol	Polyoxygenated cyclohexene	Pretreatment with 1 mg/ear deceased the xylene induced ear edema in time dependent manner.	Seangphakdee et al., 2013
*Xylopiadiscreta* (L.f) Sprague & hutch	Leave and seed	Crude leaf methanol extract	–	IL-12, TNF α, and IL-10 production was deceased in leishmania infected macrophages with a Sensitivity index of 64.8 J774 cells.	López et al., 2009
*Xylopia aethiopica* (Dunal A.Rich)	Fruit	Ethanolic extract and xylopic acid	–	Pretreatment with 300 mg/kg significantly reduced by 49.84 ± 3.94 and 43.62 ± 1.01%, respectively	Woode et al., 2012b; Obiri and Osafo, 2013
	Dried Fruit	Water extract	–	Potent antioxidant activity	Odukoya et al., 2005
*Xylopia laevigata* (Mart.) R.E.Fr.	Leaf	Hydrodistilled oil	Leaf containing essential oils	Pre-treatment with 50 mg/kg of extract significant reduced carrageenan-induced peritonitis and carrageenan induced hindpaw edema in mice	Queiroz et al., 2014
*Xylopia langsdorffiana* St Hilaire and Tulasne	Leaves	Ethanolic and hexane extract	–	Pre-treatment with 50 mg/kg produced gastroprotective effect by inhibiting the production of NO from 85 to 24%	de Albuquerque Montenegro et al., 2014
*Xylopia parviflora* Spruc	Seeds	Water, ethanolic, and hydroethanolic extracts	–	Highest inhibition of LDL oxidation and NO scavenging activity was observed at a concentration range of 250–500 μg/ml	Kuate et al., 2011
	Fruits	Hydrodistilled oil	Essential oil	A dose-dependent decrease in NO production with an IC_50_ of 7.47 μg/ml	Woguem et al., 2014

### Nuclear factor kappa B (NF-κB) inhibition

NF-κB is a principal transcription factor involved in regulating the gene expression of more than thousand regulatory proteins, including certain pro-inflammatory cytokines. These cytokines further induce the transcription of adhesion molecules, critical in leukocyte infiltration and transmigration to the site of injury and inflammation (Barnes and Karin, 1997; Tak and Firestein, 2001; Akira et al., 2006). Early studies on NF-κB has confirmed its role in gene regulation of the Igκ light chain and numerous other regulatory genes responsible for carrying out normal physiological functions, including immune modulation, acute inflammatory response, cell differentiation and apoptosis (Baeuerle and Baltimore, 1996; Yamamoto and Gaynor, 2001a; Karin et al., 2006). Five mammalian proteins have been recognized in NF-κB activation pathway: NF-κB (p50 and its precursor p105), NF-κB2 (p52 and its precursor p100), p65 (RelA), RelB, and c-Rel. These proteins can interact with each other as homo- or heterodimers, depending upon their active or latent state. A Rel homology domain is characteristically present on all the NF-κB members, which encompasses a nuclear localization array for binding of specific DNA sequences, sites for dimerization, and interface with inhibitory IκB proteins. In the cytoplasm, these inhibitory proteins (IκBα, IκBβ, IκBε, and Bcl-3) are non-covalently bonded to the NF-κB dimer keeping it in an inactive state. Upon receiving the stimulus, 26S proteosome seeks the help of IκB kinases for the phosphorylation and polyubiquition of IκB (Karin and Ben-Neriah, 2000; Broide et al., 2005). This makes the way for promoter genes to interact with nuclear localization site and activates the transcriptional factors. This activation permits NF-κB to be translocated into the nucleus, followed by the transcription of numerous cytokines pro-inflammatory cytokines (IL-1, IL-2, IL-6, IL-12, TNFα), chemokines (IL-8, Rantes, MCP-1, MIP-1α, eotaxin), cell adhesion molecules (ICAM-1 and VCAM-1) acute phase proteins (SAA, CRP), and the inducible enzymes, nitric oxide synthetase (iNOS) and cyclooxygenase (COX-2) (Barnes and Karin, 1997; Tak and Firestein, 2001; Yamamoto and Gaynor, 2001a; Gordon and Taylor, 2005). Henceforth, NF-κB activation can be inculpated for the production of numerous immunomudulators, responsible for many inflammatory diseases. Clinical data has suggested that elevated expression of these inflammatory mediators have been observed in inflamed synovial tissues, hence leading to the development and progression of rheumatoid arthritis (Marok et al., 1996). Moreover, overexpressed COX-2 expression contributes to the prostanoids production, which further promotes the synthesis of IL-1 and TNFα, leading to chronic inflammatory diseases. Thus, the natural product with NF-κB inhibitory activity represents a potential therapeutic alternative in treating inflammation (Barnes and Karin, 1997; Yamamoto and Gaynor, 2001b).

**Figure 1:**
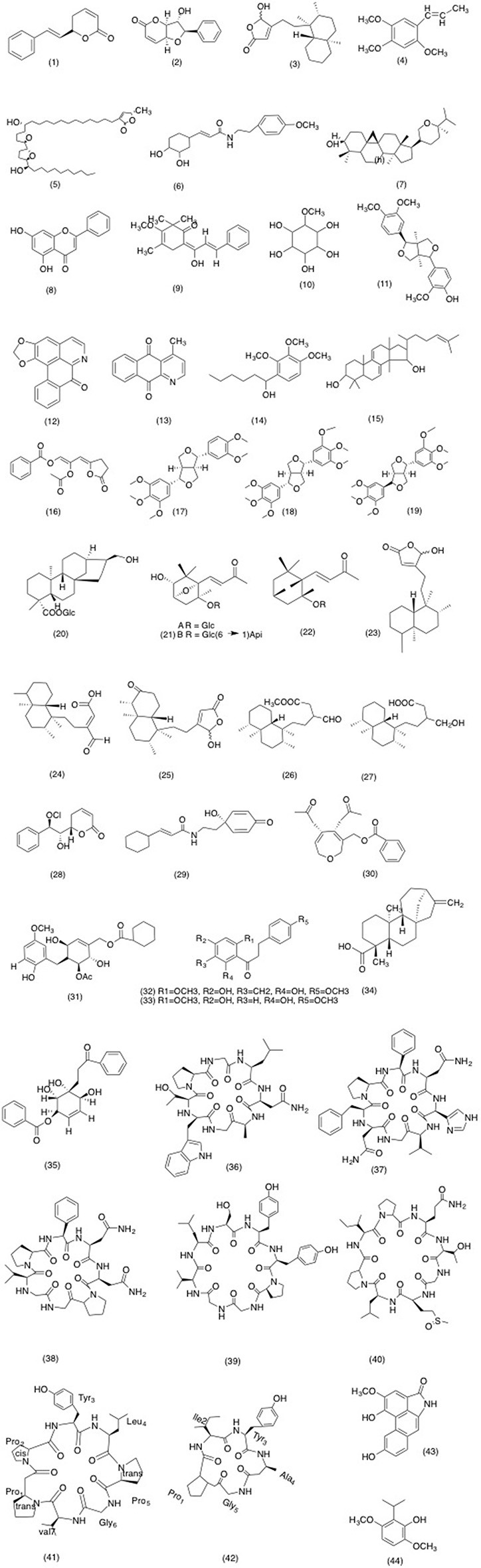
Structure of compounds isolated from Annonaceae species with potent anti-inflammatory activity (1) Goniothalamin, (2) Altholactone, (3) 6-hydroxycleroda-3,13-dien-15,16-olide (PL3), (4) alpha asarone, (5) Isodesacetyluvaricin, (6) 7′-(3′,4′-dihydroxyphenyl)-n-[(4-methoxyphenyl) ethyl] Propenamide (z23), (7) berenjenol, (8) chrysin (9) desmosdurnotin C, (10) Quebrachitol, (11) Phylligenin, (12) liriodenine, (13) Cliestopholine, (14) dehydroanonaine 1-(2′, 3′, 4′-Trimethoxyphenyl)hexan-1-ol, (15) Polycarpol, (16) acetylmelodorinol, (17) magnolin, (18) epiyangambin, (19) yangambi, (20) 7 beta, 17-dihydroxy-ent-kaur-15-en-19-oic acid 19-O-beta-D-glucopyranoside ester, (21) Milbasides A and B, (22) Milbasides C, (23) 16-hydroxycleroda-3,13-dien-15,16-olid, (24) 16-oxocleroda-3,13-dien-15-oic acid, (25) 16-hydroxycleroda-13-ene-15,16-olide-3-one, (26) 16-oxocleroda-3,13(14)*E*-dien-15-oic acid methyl ester, (27) 16-hydroxycleroda-3,13(14)*E*-dien-15-oic acid, (28) (28) Parvistones, (29) melodamide A, (30) 3-methyl-4,5-dihydro-oxepine, (31) flexuvarol B, (32) 4′6′-dihydroxy-2′,4-dimethoxy-5′-(2″-hydroxybenzyl) dihydrochalcone, (33) dihydrochalcone, 4′,6′-dihydroxy-2′,4-dimethoxydihydrochalcone, (34) Kaur-16-en-19-oic acid, (35) Zeylenol, (36) Cyclomontanins A (37) Cyclomontanins B (38) Cyclomontanins (39) Cyclomontanins D (40) cherimolacycopeptide B, (41) fanlizhicyclopeptide B, (43) Aristololactam.

Numerous Annonaceae species have been investigated for the inhibitory activity on NF-kB pathway. For example, styryl lactones from the genus *Goniothalamus* are secondary metabolites with either 5-or 6-membered lactones (De Fátima et al., 2006) with several reported bioactivities including cytotoxicity, apoptosis, and anti-inflammatory (De Fátima et al., 2006; Kuo et al., 2011). In recent work of Orlikova et al. (2013), goniothalamin (1) (GTN) (Figure 1) a styryl-lactone isolated from the *Goniothalamus macrophyllus* was evaluated on TNF-α induced NF-κB activation. GTN inhibited the TNF-α induced NF-κB activation in K562 chronic myelogenous leukemia cells at a concentration of 5 μM. Moreover, GTN also prevented NF-κB binding with its DNA transcription factors. Translocation of the p50/p65 heterodimer to the nucleus was down regulated and TNF-α activated interleukin 8 (IL-8) expression was also significantly reduced. Furthermore, in a phytochemical evaluation of Indonesian plant species, methanolic extract of *Alphonsea javanica* showed potent anti-inflammatory activity by inhibiting NF-κB activation in raw macrophage 264.7 cells (Johnson et al., 2013). Moreover, IκBα phosphorylation was also significantly reduced. This phytochemical analysis suggested that reported activity was due to the presence of an styryl lactone, altholactone (2) (Figure 1). However, according to Taha et al. (2014) methanolic bark extract of *Pseuduvaria monticola* have shown insignificant results in anti-diabetic evaluation studies. Other parameters including, NF-κB translocation were also evaluated on pancreatic insulinoma cells of mice. Test extract failed to show inhibitory activity against TNF induced NF-κB translocation. However, Shih et al. (2010) reported a completely different anti-inflammatory activity of *Polyalthia longifolia*. Since microglia mediated inflammation is involved in the pathway responsible for neuronal cell death in neurodegenerative diseases (Gebicke-Haerter, 2001). Hence, this study was carried out to evaluate the effects of *P. longifolia var. pendul* isolate, 6-hydroxycleroda-3,13-dien-15,16- olide (3)(PL3) (Figure 1) on LPS induced microglial inflammation. PL3 (3) successfully decreased the cell viability in neuroblastoma SH-SY5Y cells. Subsequently, it decreased the activity of NF-κB and the degradation of IκBα. PL3(3) also boosted HO-1 expression, which is a known cytoprotective and anti-inflammatory enzyme. Moreover, microglial activation is also associated with the complete or partial loss of dopaminergic neurons in Parkinson's disease (PD) (McGeer and McGeer, 2004; Suzumura et al., 2006; Block et al., 2007). Hence, if early measures are taken to suppress the microglial activation, then it would be an important step in suppressing the progression of PD. α- asarone (4) (Figure 1), an active constituent found in few Annonaceae species, has proved to beneficial in the early treatment of PD (López et al., 1993; Silva et al., 2007; Kim et al., 2015). Kim et al. (2015) reported that α-asarone(4) decreased the pro- inflammatory cytokine production in LPS induced BV-2 cells. The detailed mechanistic study revealed that reported activity of α-asarone was due to the inhibition of NF-κB, by blocking degradation of Ik-B signaling in BV-2 microglial cells. Moreover, in a recent study it was suggested that methanolic extract of *A. reticulata* have shown significant neuro-protective response in H_2_O_2_ induced neuronal damage in SHSY5Y cells and DRG neuronal cells. After the completion of the drug treatment the levels of pro-inflammatory cytokines, iNOS, and NF-κB activation was significantly reduced in a dose dependent manner. Hence it was concluded from this study methanolic extract of *A. reticulata* has potential to inhibit neuronal inflammation, neurogenic pain, and oxidative stress by inhibiting NF-κB inflammatory pathway (Kandimalla et al., 2017).

NF-κB activation is accountable for the development and progression of solid and hemopoietic malignancies (Van Waes, 2007; Yang F. et al., 2012). Therefore, NF-κB is often referred as an oncogene as well. Although, NF-κB activation is not a pre-requisite for tumor, but it plays an important role in moderating inflammation, setting up the tumor microenvironment and stimulating immunomodulatory cells including, pro and anti-inflammatory cytokines, and chemokine production (Mantovani et al., 2008, 2010; Grivennikov et al., 2010). Here, two pathological conditions have been stated as examples to signify the role of NF-κB activation in inflammation leading to cancer. The first example is Colitis-associated colon cancer (CAC) a classical inflammation-driven cancer and, secondly, hepatitis C induced hepatic cancer is the most prominent examples of inflammation leading to cancer (Greten et al., 2004; Pikarsky et al., 2004). Henceforth, the agents with the tenacity to subdue the NF-κB translocation are of major interest due to their dual action on inflammation and cancer development (Zhang et al., 2005; Opferman, 2008; Cheng et al., 2012). Moghadamtousi et al. (2014a) evaluated the ethyl acetic acid extract of *A. muricata* (leaves) (AMEAE) on A549 lung cancer cells. Cell viability study demonstrated the specific cytotoxic impact of AMEAE toward A549, with an IC_50_ of 5.09 ± 0.41 μg/mL after 72 h of treatment. In addition, AMEAE also inhibited the translocation of NF-κB from the cytoplasm to nucleus. Apropos to last study reported, Pieme et al. (2014) suggested that the presences of phenolic compounds are the key elements for the reported NF-κB activity of *A. muricata* (Duraipandiyan et al., 2006; Jiménez et al., 2014; Solomon-Wisdom et al., 2014).

### Prostaglandin (PGs) inhibition

The association between the PGs and inflammation was acknowledged in 1971. While two research groups reported that inhibiting the PGE_2_ production through COX enzyme is responsible for the anti-inflammatory activity of Asprin (Smith and Willis, 1971; Moncada et al., 1976). Later the discovery of two different isoforms of COX enzyme COX 1 and COX 2 further highlighted the role of PGs in inflammation. COX-1 is constitutive, expressed on platelets and gastric partial cells, responsible for modulating platelet aggregation and maintaining the gastric mucosal protective lining. Whereas, COX-2 is an inducible enzyme with cell-specific distribution. COX-2 activation give rise to prostaglandins responsible for producing classical sign and symptoms of inflammation including, hyperalgesia and swelling (Harrington et al., 2008; Rouzer and Marnett, 2009). Numerous internal and external factors including stress growth factors, mitogens, and inflammatory cytokines may cause the up regulations of COX-2 gene expression. This up regulation may give rise to development and progression of chronic inflammation, angiogenesis, and cancer metastasis (Williams et al., 1999; Dannenberg et al., 2001). Henceforth, natural products with COX-2 inhibitory activity and downregulation of over expressed COX-2 expression can be a noteworthy pharmacological therapeutic options for treatment inflammatory conditions.

Annonaceous acetogenins are usually 35–77 carbon compounds, produced by polyketide pathway (Liaw et al., 2010). First acetogenins was discovered in 1982 and ever since series of investigation has been carried on this fascinating class of natural product due to its unique structure and versatile bio-activities (Chang and Wu, 2001; Kojima and Tanaka, 2009; de Sousa et al., 2010; Chen et al., 2012; Zhang et al., 2015). In the quest to explore phytochemicals with COX 2 inhibitory activity, isodesacetyluvaricin (5) (Figure 1), an annonaceous acetogenin from *Annona glabra* was evaluated on A431 carcinoma cells (Wu et al., 2012). The addition of 25 μg/mL of Epidermal Growth Factor (EGF) resulted in increased expression of COX-2 mRNA, without effecting COX-1. Upon addition of isodesacetyluvaricin (5), expression of COX-2 mRNA was significantly reduced in a dose dependent manner, without affecting COX 1 expression. Upon further investigation, it was proposed that selective COX-2 inhibition was due to the suppression of promotor activity of element binding factor (CREB) and the nuclear factor of activated T cells (NFAT) responsible for the EGF-mediated transcriptional activation of COX-2 (Duque et al., 2005; Yiu and Toker, 2006). Isodesacetyluvaricin(5) significantly inhibited (*P* < 0.05) CREB and NFAT at a concentration ranging 1–5 μM further cementing the proposed mechanism. However, an alternative mechanism was also proposed, involving the phosphorylation of CREB by protein kinase A- dependent Rap1-extracellular-signal-related kinase and dephosphorylation of NFAT (Iñiguez et al., 2000; Chun and Surh, 2004). Alkaloid (compound 1; name not specified by author) isolated from *Fissistigma cavaleriei* root showed corresponding activity (Yang Z. et al., 2012). Colorimetric screening assay revealed that compound 1 has significant angiogenic property due to its selective COX-2 inhibition. Subsequently, Hu et al. (2008) further supported the COX-2 inhibitory activity of *Fissistigma oldhamii*. 7′-(3′, 4′-Dihydroxyphenyl)-n-[(4-methoxyphenyl) ethyl] propenamide (Z23) (6) (Figure 1) isolated from the leaves of *F. oldhamii* reduced the COX 2 gene expression in LPS induced Raw 256.7 macrophage cells. Moreover, Aquila et al. (2009) reported that cycloartane triterpene, berenjenol (7) (Figure 1) isolated from *Oxandra xylopioides*, significantly (*p* < 0.05) decreased the COX-2 gene expression at 50 μM.

*Mitrella kentia* is a tree-climbing liana from Annonaceae family. It is proposed that it possesses significant anti-inflammatory activity due to presence various bioactive compounds including, isoquinoline alkaloids, chalcones, and essential oils. Saadawi et al. (2012) evaluated the PGE_2_ inhibitory activity of *M. kentii* using highly sensitive radioimmunoassay technique. Among all the isolated compounds chrysin (8) (Figure 1) showed dose dependent PGE_2_ inhibition with an IC_50_ value of 25.5 μM. Results from another report further supported the corresponding *M. kenti* activity against PGE_2_. Desmosdumotin C (9) (Figure 1) a new isolated bioactive compound from *M. kentii* revealed gastro-protective activity by decreasing gastric ulcers area, edema, and leukocyte infiltration, which could be attributed intervention with anti H pylori and COX-2 inhibitory pathway (Sidahmed et al., 2013).

### Platlet-activation factor (PAF) inhibition

PAF plays a significant role in carrying out several physiological functions. Unfortunately when body faces mechanical stress, trauma or exposed to toxin the concentrations of PAF rises significantly. These elevated levels may give rise to numerous pathophysiological conditions such as inflammation (Stafforini et al., 2003), allergy (Petersen et al., 1997), asthma (Kasperska-Zajac et al., 2008), and thrombosis (Zhang et al., 2001). Lately, it is proposed that in order to carry out its pathophysiological functions it is imperative for PAF to specifically binds to its receptors (Esquenazi and Bazan, 2010). Therefore, compounds with PAF receptor antagonistic activity can be used as a good therapeutic option to treat PAF associated inflammatory condition (Moharam et al., 2010b). Moreover, PAF and prostanoins share common pathway hence TXA_2_ may acts as a PAF mediator and produce the symptoms associated with PAF elevations (Badr et al., 1989). In addition, increased PGE_2_ synthesis was observed in PAF treated rat meningeal cells suggesting its role in the prostanoids secondary production (Arribas-Gómez et al., 1995).

A few species of Annonaceae have displayed compelling PAF receptor antagonistic activity. For instance, Jantan et al. (2005) evaluated PAF inhibitory activity of 49 methanolic extracts from 37 Malaysian indigenous plant species. Their inhibitory effects were evaluated using ^3^H-PAF as a ligand. 6 Zingiberaceae species, two *Cinnamomum* species and one Annonaceae species (*Goniothalamus malayanus)* were reported as novel PAF antagonists, as they exhibited noteworthy inhibitory effects with IC_50_ values ranging from 1.2 to 18.4 μg/mL. Furthermore, two alkaloids and aporphine alkaloids isolated from the twigs of *Mitrephora vulpine* were evaluated using ^3^H-PAF ligand model (Moharam et al., 2010b). Out of all the isolated compounds phylligenin (10) and quebrachitol (11) (Figure 1) managed to antagonize PAF receptor in concentration depended manner with IC_50_ values of 13.1 and 42.2 μM, respectively. Whereas, the phylligen have an IC_50_ value comparable to cedrol (10.2 μM), a potent PAF antagonist. In continuation to previous work Moharam et al. (2010a) focused on the essential oils isolated from five *Goniothalamus* species *(G. tapisoides, G. velutinus, G. clemensii, G. tapis*, and *G. woodii)*. Essential oil from the bark of *G. clemensii, G. woodii, G. velutinus*, and the root oil of *G. tapis* were able to show significant antagonist activity with IC_50_ values ranging from 3.5 to 10.5 μg/ml. It was proposed that the presence of sesquiterpenes and sesquiterpenoids were responsible for producing potent PAF receptor antagonistic activity. Furthermore, Nordin et al. (2012) highlighted the similar bioactivity of *E. pulchrum* first time ever. Among the extracts examined, ethyl acetate extract has shown superior antagonist activity with 85.6% inhibition. Moreover, liriodenine (12), cleistopholine (13) and dehydroanonaine 1-(2′, 3′, 4′-trimethoxyphenyl)hexan-1-ol(14) (Figure 1) isolated compounds from the same fraction were the strongest to antagonize PAF receptor with an IC_50_ values of 26.6, 50.2, and 45.4 μM, respectively. Saadawi et al. (2012) reported that polycarpol (15) and acetylmelodorinol (16) (Figure 1) isolated from *Mitrella kentii* (Bl.) have shown similar dose dependent inhibitory effects with IC_50_ values of 24.3 and 24.5 μM, respectively. Faria Lua Figueiredo et al. (1999) reported parallel activity for magnolin (17), epiyangambin (18), yangambin (19) (Figure 1) and furofuranic lignans isolated from leaves of *Rollinia deliciosa*.

### Inducible nitrous oxide (iNOS) inhibition

Numerous vascular diseases are often supplemented with inflammation, which may affect the production of peroxynitrite and protein nitration and may lead to irreversible DNA damage and apoptosis (Beckman and Koppenol, 1996; Zamora et al., 2000). Three different isoforms are involved in the production of Nitric oxide in various parts of the body. Neuronal NOS (nNOS, ^3^NOS1) is produced by both autonomic and peripheral nervous system to serve as a neurotransmitter and to support inter and intra neuronal communication. Chromosomes 12 carry the gene coding for nNOS. While, endothelial NOS (eNOS) and inducible NOS (iNOS, NOS_2_) is constitutively expressed isoenzyme principally produced from endothelial cells. Chromosome 7 and 17 are responsible for carrying an encoded gene for the iNOS and eNOS respectively. Elevated intracellular Ca^2^ levels are required for the activation of both enzymes. But unlike eNOS, iNOS bind with Ca^2+^ calmodulin more tightly with non-covalent bond hence making the deactivation of iNOS considerably difficult. This leads to the continuous and unstoppable overproduction of iNOS in the body (Alderton et al., 2001; Aktan, 2004; Pautz et al., 2010). Normally, low levels of NO are essential for the body to regulate and maintain the vascular permeability and homeostasis. However, if the production of NO exceeds the normal levels then it may lead to the pathogenesis of cardio vascular disorders such as hypertension, heart failure, and atherosclerosis (Cooke and Dzau, 1997; Albrecht et al., 2003). Inflamed human endothelium can contribute to increased iNOS activity up to three- to five-fold beyond its baseline concentrations (Zhang et al., 2007; Brovkovych et al., 2011). Moreover, numerous reports have indicated that eNOS can mimic iNOS activity depending upon the nature and intensity of the stimulus, but more work need to be done to elucidate the mechanism behind this interchangeable behaviors of eNOS (Cirino et al., 2003).

There are several reports in the literature that show that Annonaceae species has a potent iNOS inhibitory activity. Recently Hu et al. (2007) reported that 7-(3,4- dihydroxyphenyl)-N-[(4-methoxyphenyl) ethyl] propenamide (Z23) (6) (Figure 1) from *F. oldhamii* exhibited dual action of decreasing the T cell activation and the production of iNOS in *in vivo* model of type II bovine collagen induced arthritis. In the light of this study, it was suggested that anti-inflammatory effect of Z23, is through modulating the synthesis of several inflammatory mediators and cytokines involved in the inflammatory process. In conclusion, Z23 has the potential to be a therapeutic option for numerous inflammatory diseases, where the overproduction of NOS and inflammatory cytokines are responsible for development and progression of the disease, e.g., rheumatoid arthritis. Moreover, Adewole and Caxton-Martins (2006) evaluated the corresponding activity of aqueous leave extract of *A. muricata* on streptozotocin induced (STZ)-diabetic rats. The extracts were intra-peritoneally injected to the rats (100 mg/kg) starting from 5 days after the administration of STZ and stopped on the 30th day of the study period. While control group of rats was injected with same amount of citrate buffer. Histophathological evaluation and bioassay results revealed that *A. muricata* significantly (*p* < 0.05) reduced the glucose and iNOS level in a dose dependent manner. Hence in the light of this study it can be concluded that *A. muricata* has beneficial effects on pancreatic tissues subjected to STZ-induced oxidative stress. Likewise the recent work of Nhiem et al. (2015) reported three new *ent*-kaurane diterpenoids and five known isolates from the fruit extract of *A. glabra* and they were evaluated for anti-inflammatory activity. After 24 h of incubation with the isolates, 7β,17-dihydroxy-*ent*-kaur-15-en-19-oic acid 19-*O*-β-D-glucopyranoside ester (20) (Figure 1) inhibitory activity was the most significant with the IC_50_ value of 0.01 μM. Similarly, Shirwaikar et al. (2004) focused on *Annona Squamosa*, commonly known as custard apple. Several antioxidant-screening models were used to evaluate the free radical scavenging activity of the leaves of *A. squamosa*. Leave ethanolic extract had the highest scavenging activity against 2,2-azinobis- (3-ethylbenzothiazoline- 6- sulphonate) (ABTS) up to 99.07% followed by the scavenging of the stable radical 1, 1-diphenyl, 2- picryl hydrazyl (DPPH) (89.77%), and nitric oxide radical (73.64%) at 1,000 μg/ml. These findings signify the therapeutic potential of *Annona* species in traditional medicine.

*Xylopia parviflora* is a tall tree distributed in East and Central Africa. It is very famous for its traditional uses in coastal regions against stomach disorders, headaches and fever (Nishiyama et al., 2006). Kuate et al. (2011) evaluated the possibility of using *X. parviflora* seed as a food source of natural antioxidant. Several bioassays were performed on water, ethanolic, and hydro-ethanolic extracts to evaluate the anti-oxidant and free radical scavenging activity. All extracts have shown significant (*p* < 0.05) dose dependent NO inhibition, compared to the control group. However, high test NO inhibition was observed with hydo-ethanolic extract, decreasing the iNOS production upto 87% with IC_50_ range of 20–50 μM. The anti-oxidant activity of *X. parviflora* was further supported by the late work of Woguem et al. (2014). This study focused on the essential oils hydro distilled from the fruits of *X. parviflora*. In order to evaluate the anti-oxidant and anti-inflammatory activity of *X. parviflora*, LPS induced raw macrophage 264.7 cell were incubated with essential oils for 24 h. Results suggested that cells treated with *X. parviflora* essential oils have shown decreased NO production in a dose dependent manner exhibiting a potential anti-inflammatory activity.

In recent years, utilization of anti-oxidants has been significantly increased due to its positive role in the management of numerous diseases (Halliwell, 2006). *Monodora tenuifolia* have already been exploited commercially either as anti-oxidant additives or nutritional supplements (Pourmorad et al., 2006). To validate the anti-oxidant activity of *M. tenuifolia* Njoku (2007) administered diethyl ether fraction and seed extracts to rats systemically. Since the diazotization of nitrite with sulphanilamide, formation of choromophore followed by coupling with napthethylene diamine served as a marker of NO scavenging activity (Villagra et al., 2007). *M. tenuifolia* seed extract, pet- ether extract and the diethyl ether fraction were able to inhibit the formation of chormophore in a dose dependent manner. Although, the exact pathway of free radical scavenging activity was not completely understood but it was proposed that activity reported was probably due to the high occurrence of anti-oxidant vitamins and flavonoids. Moreover, no toxicity sign were observed up to a dose level of 5,000 mg/kg body weight. Moreover, 15 known and three new megastigmane glycosides were isolated from *Miliusa balansae* and were tested on LPS induced RAW 264.7 macrophage cells (Thao et al., 2015). milbaside A and B (21) and C (22) (Figure 1) were most effective among all isolated compounds, with inhibition values of 98.5 ± 1.6, 90.9 ± 7.8, 84.8 ± 3.5%, respectively. Rest of the compounds either had weak or failed to show any activity on tested concentration (10.0, 20.0, and 40.0 μM).

*Polyalthia longifolia* var. *pendula*, commonly known as “Indian Mast Tree” is widely distributed in tropical and subtropical regions. Due to its ability to reduce noise pollution it is extensively cultivated in several Asian countries, specially in Taiwan (Ghosh et al., 2008). In an effort to explore isolates from Taiwanese medicinal plants with anti-inflammatory activity, Wu T. H. et al. (2014) evaluated *P. longifolia* isolates and fruit extracts for iNOS inhibitory activity. Results from present study suggested that 16-hydroxycleroda-3,13-dien-15,16-olide (23) and 16-oxocleroda-3,13-dien-15-oic acid (24) (Figure 1) has significantly (*P* < 0.05) reduced NO production at 10 μg/mL, with 81.1 and 86.3%, inhibition, respectively. Similarly, Saha et al. (2008) conducted a study on Bangladeshi medicinal Plants (*Hibiscus mutabilis, Leucas aspera, Ixora coccinea*, and *P. longifolia)*. Ethanolic extract of all the tested species showed dose dependent NO direct scavenging activity in following manner *L. aspera* > *I. coccinea* > *H. mutabilis* > *P. longifolia*. Although, the activity reported for *P. longifolia* was least potential among all the tested extract with 70.67% with IC_50_ of 167.08 μg/ml but its inhibitory activity can be considered significant when compared to positive control (ascorbic acid with 74.56% inhibition). In another account, Johnson et al. (2013) stated that *Alphonsea javanica Scheff* decreased the expression of eight pro-inflammatory cytokines/enzymes (0.8–5.0 μM) including iNOS. Then again, Aquila et al. (2009) reported similar activity of berenjenol (7) (Figure 1), isolated from *Oxandra* cf. *xylopioides*. The anti-inflammatory activity was assessed on sub chronic inflammation induced by repetitive application of 12-O-tetradecanoyl-phorbol-13-acetate (57% inhibition, 7 × 1 μmol/ear). While it reduced the expression of iNOS by 80% at 50 μM. Hence suggesting that these species can be used as potent and novel therapeutic agent for scavenging of NO and the regulation of pathological conditions caused by excessive generation of NO and by product.

### Reactive oxygen species (ROS) inhibition

ROS are usually characterized as incompletely reduced metabolites of oxygen that have potent oxidizing potentials (Casteilla et al., 2001; Mittal et al., 2014). ROS actions can be variant depending upon their concentrations. At high concentrations, ROS are injurious to body but at low concentrations, ROS serve as intricate signaling functions (Taniyama and Griendling, 2003; DelloStritto et al., 2016). Under normal circumstances, the human body produces numerous antioxidants such as catalase and glutathione peroxidase, in order to balance out the deleterious effects of ROS. However, in certain inflammatory conditions, this balance is disturbed due to the excessive generation of ROS (Drake et al., 1998; Cominelli, 2004; Reuter et al., 2010). In other way, neutrophils produce a large quantity of ROS at the site of inflammation in order to fight against the foreign bodies. As a result lipid peroxides are produced which gives rise to pathophysiological changes associated with an oxidative stress (Wolfreys and Oliveira, 1997; Dabrowski et al., 1999; Aprioku, 2013). This physiological response is known as oxidative burst. It provides support to host defense, but it can also result in collateral destruction of host tissues (Chen and Junger, 2012). Henceforth, suppression of the excessive pathophysiological activation of neutrophils can be used to treat inflammatory diseases. A few studies including Barreca et al. (2011) evaluated the antioxidant activity of ethanolic, methanolic, and dimethyl formammide extracts of *Annona cherimola* on hydrogen peroxide induced lymphocytes. Although, all extracts showed significant antioxidant and ferric reducing potential but highest scavenging activity was reported for dimethyl formammide extract against DPPH, ABTS and ${\text{O}}_{2}^{-}$. Whereas ethanolic extract was reported to have highest activity against *tert*-butyl hydroperoxide induced lipid peroxidation. Invariably in the light of the fact that various antioxidants also possess antimutagenic activity, Ravikumar et al. (2008) focused on screening antimutagenic and antioxidant activity of *Polyalthiam cerasoides*. Antioxidant activity was evaluated based on inhibitory activity on hydroxyl radical, superoxide radical, DPPH free radical scavenging and Fe^3+^reducing properties. Results suggested that methanolic extract inhibited DPPH and superoxide anion in a dose dependent manner. Moreover, similar activity was reported against hydroxyl radicals produced by the reaction of Fe^3+^-EDTA together with H_2_O_2_ and ascorbic acid. In addition, by reducing Fe^3+^ to Fe^2+^ ions the methanolic extract had exhibited strong reducing potential. These results clearly indicate that methanolic extract of *P. cerasoides* have powerful anti-oxidant and reducing potential. Likewise, Hwang et al. (2009) reported similar activity of isopedicin, a flavanone derived from *F. oldhamii*. Results suggested that isopedicin decreased superoxide anion production in FMLP stimulated RAW macrophage with an IC_50_ value of 0.34 ± 0.03 μM. In addition, phosphodiesterase inhibition enhanced the activity of PKA and cAMP. Moreover, FMLP induced kinase and c-Jun N-terminal kinase phosphorylation was inhibited by isopedicin. However, isopedicin was unable to reverse the FMLP induced calcium mobilization and p38 mitogen activated protein kinase phosphorylation. Hence it is proposed that antioxidant activity of isopedicin is due to the elevation of cellular cAMP and activation of PKA through its inhibition of cAMP-specific PDE. Numerous other species of Annonaceae can produce similar super oxide anion generation and elastrase inhibitory activity in micro-molar concentration. For instance, Chang et al. (2006) isolated a novel clerodane diterpenoid 16-hydroxycleroda-13-ene-15,16-olide-3-one (25) (Figure 1) along with other known 23 compounds from the methanolic extract of *P. longifolia* leaves. Anti-inflammatory activities of isolated compounds were evaluated on formyl-L-methionyl-*L*-leucyl-*L*-phenylalanine/cytochalasin B (FMLB/CB) stimulated superoxide generation in neutrophils. 16-oxocleroda-3,13*E*-dien-15-oic acid methyl ester (26) and 16-hydroxycleroda-3, 13(14)*E*-dien-15-oic acid (27) (Figure 1) exhibited significant inhibitory activity against both models with IC_50_ value of 0.6 ± 0.09 and 1.49 ± 0.28 μg/mL, respectively. In continuation of previously mentioned work by Liou et al. (2014), member of same research group investigated phyto constituents from the leaves of *Polyalthia parviflora*. Parvistones (28) (Figure 1), a styryllactones and two 6S configuration derivative inhibited fMLP/CB-induced superoxide anion generation and elastase release. Likewise, Chan et al. (2013) isolated a new melodamide A(29) (Figure 1) phenolic amide along with 12 known compounds from the methanolic leave extract of *Melodorum fruticosum*. Melodamide A (29) has shown significant inhibitory activity with IC_50_ value of 5.19 μM against both O^−2^ and elastase. However, synthetic derivative of melodamide A(29) with a 2-bromo substitution on ring A failed to show any substantial anti-oxidant activity. Similarly, in recent work of Hsu et al. (2016) seven compounds including 3-methyl-4, 5-dihydro-oxepine (flexuvaroxepine A) (30) (Figure 1), four polyoxygenated cyclohexene and two polyoxygenated cyclohexene derivatives, together with four known flavones were isolated from methanolic extract of *Uvaria flexuosa*. All isolated compound were evaluated against superoxide anion generation and elastase release. Out of all the isolated compounds flexuvarol B (31) and chrysin (8) (Figure 1) have shown significant inhibitory activity against elastase release and superoxide anion generation with IC_50_ of 2.25–5.55 μM. Similarly, Njoku (2007) reported anti-oxidant activity for the seed extract *of M. tenuifolia*. He reported that seed extracts were able to inhibit lipid peroxidation and free radical generation in liver homogenate in a dose dependent manner.

### Suppressing the transmigration and phagocytosis of mono nuclear cells

More than few reports have called attention to other mechanism related with the flavonoids of *A. dioica*. This study focused on the several aspects including anti-proliferative, antioxidant, and anti-inflammatory activity of *A. dioica* (Formagio et al., 2013a). DPPH assay was employed in order to evaluate the free radical scavenging activity of four fractions including hexane, chloroform, ethyl acetate and hydromethanol fraction. Furthermore carrageenan induced paw edema test was used for the further evaluation. Results suggested that ethyl acetate and hydromethanol fractions were most potent among all the tested fractions with an IC_50_ of 8.53 and 10.57 μg/mL, respectively. Whereas, methanolic extract was able to significantly reduce the carrageenan-induced edema in dose and time dependent manner (30–300 mg/kg). It was concluded that *A. dioica St*. and several other species of Annonacea family have a unique mechanism of suppressing the transmigration and phagocytosis by polymorphonuclear leukocytes (PMNs) and helps in the alleviation of unnecessary and deleterious production of ROS, hyperalgesia, and other classical symptoms of inflammation. In succession of his previous work, Formagio et al. (2013b) further evaluated the essential oils, hydrodistillated form the leaves of *Annona sylvatica*. Carrageenan induced paw edema test was employed to assess the anti-inflammatory activity. Upon oral administration of essential oils, potent anti-inflammatory and antioxidant activity was reported. It was proposed that the presence of z-caryophyllene (44) and β-maliene (46) (Figure 1) in essential oil was responsible for potential anti-oxidant activity. Moreover, several other phytochemical classes including chalcone have been reported to share similar activity. Somsrisa et al. (2013) managed to isolated one new dihydrochalcone derivative 4′, 6′-dihydroxy-2′,4-dimethoxy-5′-(2″-hydroxybenzyl)dihydrochalcone (32) and one known dihydrochalcone, 4′, 6′-dihydroxy-2′,4- dimethoxydihydrochalcone (32) (Figure 1) from the twig and leaves of *Cyathostemma argenteum*. Ethyl phenylpropiolate was applied tropically on the inner and outer part of the ear of rats to induce ear edema. Dose of 1 mg/ear test compounds were also applied tropically just before the application of irritant to evaluate their analgesic activity. A significant time dependent inhibition was observed for both isolated compounds at a dose of 1 mg/ear. Hence concluding that both compounds are very effective in an acute phase of inflammation and has the ability to antagonize or decrease the vascular permeability of inflammatory mediators including histamine, serotonin, bradykinin, and prostaglandin (PGs). Chavan et al. (2012) reported similar activity for a diterpines, kaur-16-en-19-oic acid (34) (Figure 1) isolated from the bark of *Annona reticulate*. Hot plate method was employed to assess the analgesic activity. Whereas anti-inflammatory activity was evaluated using carrageenan induced rat paw edema assay. Significant analgesic and anti-inflammatory activity was reported for kaur-16-en-19-oic acid (34), at doses of 10 and 20 mg/kg. This study points out a lead anti-inflammatory compound, which should be further explored for therapeutics.

In the last few decades exceptional progress has been made for the development of new anti-inflammatory and analgesic drugs. However, we are far behind from finding an ideal class of drug with maximum efficacy and minimum side effects. As a part of this effort Vendramini-Costa et al. (2014) evaluated a styryl-lacton, goniothalamin (1) (Figure 1) widely distributed among the genus *Goniothalamus*. Results suggested that *Goniothalamus* has significantly reduced the carrageenan induced paw edema in mice. Moreover, effective concentrations were also evaluated for its potential toxicity and results suggested that no sign of toxicity was observed at effective concentrations. Moreover, *Meiocarpidium lepidotum* has exhibited significant analgesic and anti-inflammatory activity in mice and rat (Meddah et al., 2013). Tail flick tests, acetic acid- induced writhing, carrageenan-induced hyperalgesia in mice were employed for the evaluation of anti-inflammatory activity. Paw edema was significantly reduced after the administration of extract. Moreover, acetic acid induced writhings and tail-flicks were significantly (*p* < 0.001) reduced at 1 mg/kg dose. Hence, proving a very potent analgesic and anti-inflammatory activity at small concentrations. Moreover, the intra-peritonial injection of methanolic fruit extract of *D. chrysocarpa* in rat model have shown significant analgesic and anti-inflammatory activity in a dose dependent manner (Almeida et al., 2012). At dose range of 100, 200, and 400 mg/kg acetic-acid-induced abdominal writhes were significantly reduced. Moreover, significant results were also recorded for hot-plate test and formalin test. It was proposed that corresponding activity reported is due to its dual action on peripheral and central nervous system. Additionally, Ishola et al. (2016) reported similar activity for hydro-ethanolic seed extract of *Monodora myristica*. It completely inhibited the xylene induced ear edema. Significant increase in threshold and decrease in acetic induce abdominal writings were observed in a dose dependent manner at concentration range of 50–200 mg/kg. Hence, this study provides scientific evidence for the use of *M. myristica* in traditional medicine for pain management. In the latest work of Popoola et al. (2016) focused three plant species commonly used for their indigenous anti-cancer activity. In this present study anti-oxidative and anti-inflammatory activity of *Garcinia kola Heckel* (stem bark), *Uvaria chamae* (root), and *Olax subscorpioidea* (root) were evaluated using *in vivo* inflammatory models. Formaldehyde and carrageenan induced rat paw edema was significantly reduced by all three species, in a time dependent manner. Maximum inhibitory activity was observed at 400 mg/kg when compared with the reference drugs. Thus, this study provides some scientific evidence of the usage of these three species in traditional anti-cancer and anti-inflammatory regimens. Seangphakdee et al. (2013) reported similar anti-inflammatory activity for poly-oxygenated cyclohexane zeylenol (35) (Figure 1) isolated from *Uvaria grandiflora*. Anti-inflammatory activity of zeylenol (35) was evaluated on rats using ear edema assay. Zeylenol (35) reduced the ear edema in a time dependent manner; activity recorded was equivalent to positive control, phenylbutazone. Results suggested that the test compound is effective against acute phase inflammation and able to inhibit the synthesis or release of various inflammatory mediators (histamine, serotonin, bradikinine, prostaglandin) responsible for producing vasodilation. Moreover, the mice treated with essential oil with the leave of *Xylopia laevigata* have also significantly (*P* < 0.05 and *P* < 0.001) reduced formalin induced abdominal writhing (Queiroz et al., 2014). Whereas, oral administration of essential oils decreased carrageenan-induced peritonitis and paws edema. Hence providing the scientific evidence to the traditional use of *X. laevigata* as anti-inflammatory remedy.

### Pro-inflammatory cytokines inhibition

According to Bulua et al. (2011) “mitochondrial ROS (mtROS) act as signaling elements to induce pro-inflammatory cytokine production” (Nakahira et al., 2011; Zhou et al., 2011). This self-explanatory statement highlights the role of ROS in the production of pro-inflammatory cytokines. TNF-α is a major cytokine, responsible for inducing various other proinflammatory cytokines. Using its pyrogic activity TNF- α can induce mononuclear cells to produce inflammatory mediators like iNOS and ROS. These inflammatory mediators can further induce the production of TNF- α simultaneously. This leads to the production of IL-1 and IL-6 and proinflammatory cytokines and chemokines by activation of NF-κB (De Simone et al., 2015; Chen et al., 2016). Tumor necrosis factor alpha (TNF-α) and interleukin-6 (IL-6) are pro-inflammatory cytokines released by stimulated macrophages to augment the inflammatory response and injure cells and its surroundings (Whiteley et al., 2009; Olefsky and Glass, 2010). Hence decreasing the TNF-α and IL-6 levels may repress tissue injury caused by the inflammation. For instance fruit and seed extract from *A. squamosa* decreased TNF-α and IL-6 levels in LPS-stimulated macrophages (Yang et al., 2002). Similar activity was reported by Chuang et al. (2008) for *Annona montana*. The study was conducted to evaluate four new cyclomontanins isolated from the methanolic extract of *A. montana seeds*. LPS induced murine macrophage J774A.1 cells were used to evaluate the cytokines production inhibitory activity of cyclomontanins. Results suggest that maximum TNF-α and IL-6 inhibitory activity was reported for cyclomontanin A (36) and cyclomontanin C (37) (Figure 1). Although, cyclomontanin D (38) and annomuricatin C (37) (Figure 1) inhibitory activity is at relatively higher concentration (30 μg/mL). However, upon stimulating the cells with Pam3Cys cyclomontanin D exhibited dose dependent inhibition at various concentrations 3, 5, 10, 30, and 50 μg/mL. These results demonstrated a potent anti-inflammatory activity of synthetic analogs of cyclopeptides but lacks accuracy to pin point the exact mechanism of inhibition. Dellai et al. (2010) reported similar activity of cyclic peptides isolated from the seeds of *A. squamosa*. cyclosquamosin D (39) and met-cherimolacyclopeptide B (40) (Figure 1) and their analogs were evaluated using well established enzyme-linked immunosorbent assay (ELISA). Fourteen synthetic analogs were prepared from cyclosquamosin D (39) (Figure 1), few of them failed to show any bioactivity and hence they were included as a negative control. Whereas, few synthetic compounds were able to show superior activity then the natural products. Three analogs were able to suppress the IL-6 and TNF α equally. Moreover, no activity was reported for the natural cyclic peptide cherimolacyclopeptide B and met-cherimolacyclopeptide. Whereas, their analogs were able to significantly reduce the production of TNF-a and IL-6 in LPS induced macrophage cells. Two new cyclic peptides, fanlizhicyclopeptide A (41) and fanlizhicyclopeptide B (42) (Figure 1) isolated from the fruit of *A. squamosa* reported similar activity (Wu P. et al., 2014). Fanlizhicyclopeptide A (41) and fanlizhicyclopeptide B(42) (Figure 1) were able to reduce the secretion of TNF α by 32 and 27%. However, IL-6 activity was more pronounced then TNF α with 51 and 57% inhibition. Few years later Ge et al. (2013) demonstrated that aristololactam (43) (Figure 1) an alkaloid extracted from *F. oldhamii* against TNF-a and IL-6. From the results, the ethanol extract and its CHCl_3_, EtOAc, and n-BuOH-soluble parts showed significant inhibitory effects against LPS-induced IL-6 production, and the CHCl_3_-soluble part inhibited TNF- α production. As mentioned before 7′-(3′, 4′-dihydroxyphenyl)-n-[(4-methoxyphenyl) ethyl] propenamide (Z23) (6) (Figure 1) from *F. oldhamii*, have shown significant inhibitory activity against nitric oxide synthase (iNOS) and cyclooxygenase 2 (COX2). In the same study, Hu et al. (2008) reported that Z23 was able to produce more pronounce effect on TNF- α then IL-6. Moreover, in recent study of Vendramini-Costa et al. (2017) evaluated the cytokines inhibitor activity of goniothalamin (1) in colitis-associated cancer (CAC) and dextran sulfate sodium (DSS) induced-colitis mice model. Results indicate that goniothalamin decreased the gene expression of IL 1β, TNF-α, IL-6, IL-23A, IL-22, and IL-17A. Moreover, IL-6, IL-17, and TNF-α production was also significantly reduced in tumor tissue. It was concluded from this data that GTN could be a potential candidate for the treatment of progression and development of colon rectal cancer due to its potent anti-inflammatory activity. Rojano et al. (2007) indicated one new and unusual cycloartane triterpene, berenjenol (1) (Figure 1) and its three synthetic derivative isolated from the leaves of *Oxandra xylopioides*. Test compounds were co-incubated with LPS induced RAW 264.7 macrophages. Whereas, the inhibitory activity of the test compound was evaluated using ELISA kit. Isoespintanol (Figure 1), a synthetic derivative of berenjenol (1) had decreased IL-1 production by 72% at 100 μM and reduced IL-1 mRNA synthesis.

In very interesting study conducted by López et al. (2009), reported anti leishmanial and anti-inflammatory activity of eight extracts and essential oil from the leaves and seeds of Xylopia discrete. Results from this study suggested that *Xylopia discrete* failed to produce any significant effect on IL-10 IL-12 and TNF α. However, rise in level of monocyte chemotactic protein 1 (MCP-1) was observed in infected macrophages. This rise of MCP-1 caused to decrease the number of Leishmania parasites in infected cells. The proposed mechanism involves modulating the levels of MCP-1, which is responsible for the prophylactic function in maintaining cytokines level (Brandonisio et al., 2002; Dey et al., 2007). Usually, macrophage produce pro-inflammatory cytokines plus MCP-1 to induces the production of IL-12 and inhibiting IL-10 and TGF-β. Th1 phenotype was provoked in response to treatment with MCP-1, macrophage inflammatory protein supports prophylactic role to these chemokines to control of Leishmania parasites. This study suggests that *X. discreta* possesses antileishmanicidal potential due to its immunomodulatory activity. But more work need to be done *in vivo models* in order to confirm these bioactivities.

## Clinical trials

Currently, only one double-blind randomized, placebo-controlled, Phase 0 trial clinical trial has been carried out to highlight the anti-inflammatory effect of Annonaceae sp. http://www.clinicaltrials.gov/. The purpose of this study was to evaluate *A. muricata* for its nutritional status, role in improving the quality of life, and effect on fecal butyrate, inflammation, and colorectal cancer cells. The leaves of *A. muricata* were focused in this study due to the presences of biologically active polyphenols and acetogenins with reported anti-inflammatory and anti-cancer activity. Moreover, this was an effort to validate the previous findings in *in vitro*, animal study and traditional uses of this specie. Thirty patients were involved in this study. Both genders were included with the age of 18 years and above. Patients with satisfactory hematological reports and Karnofsky performance >60% were taken into the study. Two patient groups were formed; one group was treated with crude ethanolic extract of *A. muricata* while other group served as a control group (maltose treated group). After every fortnight patient's dietary intakes were assessed. Whereas, the hematological reports, fecal butyrate level, nutritional status, and markers of system systemic inflammation of the patients were assessed in the beginning and the end of the study. *A. muricata* ethanol extract were administered with 300 mg/day dose, whereas equal amount of cellulose was administered to control group for 8 weeks to evaluate anti-inflammation and anti-proliferative activity. Whereas, the ethanol-soluble fraction of water extract was used along placebo to evaluate the nutritional value of *A. muricata*. Primary outcome measures after 8 weeks of the study concluded that *A. muricata* has nutritional status and can be used for as a dietary supplement to improve the quality of Life. Secondary outcome measures stated that extract of *A. muricata* did not show any cytotoxic activities in MTT assay using colorectal cells when exposed to patient serum for 48 h. Nevertheless, no data was provided regarding anti-inflammatory activity of the extract, neither material nor methods were discussed in detail. Therefore, this interesting study may lead to insightful development of knowledge regarding its clinical efficacy. Since the number of patients participated in this clinical trials were insufficient and duration of study should be more than 8 weeks, and results of parameters under investigation were not discussed properly and left open-ended. Nonetheless, more randomized controlled trials are required to cover additional parameters to draw fruitful conclusion about *A. muricata* and other Annonacaee species.

## Toxicology

Numerous studies have been conducted to explore the pharmacological properties of Annonaceae sp. While the toxicological aspects associated with the use of these species have been ignored significantly. However, according to toxicological studies the frequent use of few Annonaceae species has been associated with potentially hazardous side effects. For instance, Lannuzel et al. (2002) carried out the study to evaluate the abnormally high prevalence of levodopa-resistant Parkinsonism in West Indies. Some proposed that frequent consumption of fruit of *A. muricata* is the reason behind this atypical Parkinsonism. To validate this claim mesencephalic dopaminergic neurons were exposed to alkaloids and root and bark extracts (totum) of *A. muricata* for 24 h. After 24 h it was observed that 50% of dopaminergic neurons were deteriorated with, 4.3 μg/ml (13 μM) coreximine, or 100 μg/ml (304 μM) reticuline and 18 μg/ml totum. Microscopic visualization of dead neuron revealed DNA fragmentation, purposing apoptosis to be the possible cause of death. A few years later Champy et al. (2004) shared the similar concern toward the inhabitants of Guadeloupe island. He proposed that atypical Parkinsonism is prevalent in this region due to frequent consumption of the annonaceous acetogenins (lipophilic complex I inhibitors) present in *A. muricata*. To elucidate this claim, high concentrations of annonacin (3.8 and 7.6 mg per kg per day for 28 days) were intravenously administered to rats. Annonacin inhibited brain homogenates complex 1 in concentrations dependent manner and ATP level inside brain was reduced by 44%. Although, no systemic toxicity was observed but neuro-pathological abnormalities were observed in the basal ganglia and brainstem nuclei. Significant loss of dopaminegernic (−31.7%), cholinergic (−37.9%), GABAergic neurons (−39.3%) in the stratum was accompanied by increase numbers of astrocytes cell (35.4%). Present findings are enough to support the hypothesis that annonacin may be involved in the Guadeloupean Parkinsonism and bolster the theory that ecological toxins may instigate atypical Parkinsonism. Lannuzel et al. (2003) reported annonacin to be more toxic than 1-methyl-4-phenylpyridinium (MPP^+^) (EC_50_ 0.018 vs. 1.9 μM) in dopaminergic neuronal dysfunction. It was proposed that Annonacin interfares with the energy production mechanism of neuronal cells hence leading to the dopaminergic neuronal cell death. These studies conclude that these species may alter functions of dopaminergic nerve cells *in vitro*. It is in this way possible that *A. muricata* could bring about the neuronal dysfunction and neuro degenerative diseases upon frequent use (Caparros-Lefebvre and Lees, 2005; Ludolph et al., 2009).

*A. congensis* bark and *X. aethiopica* fruits have been extensively used in the treatment of diabetes and due to its extensive use in tropical regions Ogbonnia et al. (2008) designed this study to evaluated the acute and subacute toxicity of a water: alcoholic extract (1:1) on Swiss albino rats. Animals were fed with various concentrations ranging from 1 to 20 g/kg body weight for 30 days. Significant amount of weight gain was observed at low doses but there were no signs of drug induced toxicity or animal death at these concentrations and. However, sub-acute toxicity study have shown signs of renal toxicity. Moreover, an acute and sub-acute toxicity study was conducted on the aqueous stem-bark extract of *E. chlorantha* (Tan et al., 2007). Acute toxicity was evaluated by administering single oral dose of 1,000, 3,000, and 5,000 mg/kg of plant extract to rats and monitoring for any signs of growth impairment and death for 7 consecutive days. Sub-acute toxicity was assessed by evaluation of hematological and biochemical parameters after administrating 250, 500, and 1,000 mg/kg extract for 42 consecutive days. Rats were sacrificed on 42nd day to study the histological analysis of vital organs including heart, lungs, liver, kidney. At acute doses no death, drug induced symptoms or growth impairment was observed. Whereas, sub-acute toxicity study presented histopathological signs in the liver, lungs, and kidneys at dose of 1,000 mg/kg. Moreover, significant (*P* < 0.05) increase in values of ALT, AST, and platelet counts were also observed. Hence concluding that the *E. chlorantha* extract exhibit no acute toxicity up to 5,000 mg/kg, but can cause lung, hepatic and kidney disorders at doses >500 mg/kg. Moody et al. (2007) in his recent study evaluate the acute and sub-chronic toxicity of *E. chlorantha*. Ethanolic extracts were administered to Swiss rats through oral and intra-peritoneal route of administration. Upon histo-pathological examination, no pathological signs were observed on any organ expect for lungs, which exhibited mild to moderate edema upon examination.

## Conclusions and future directions

This review is an effort to abridge the ethnobotany, morphology, phytochemistry, and particularly focusing on the anti-inflammatory activity of the Annonaceae species. It additionally conveys insights of the Annonaceae family, which may contribute toward highlighting its isolated compounds as a future candidate for drug discovery. A careful review of the literature has shown that few studies have proposed the scientific evidence for the traditional uses of Annonaceae sp., its pleotropic therapeutic activities including analgesic, anti-pyretic, anti-ulcer, anti-hypertensive, oral hypoglycemic and wound healing. Sesquiterpenes and diterpenes from Annonaceae have shown promising anti-inflammatory activity. Hence making this class of drug potential clinical trial candidates in anti-inflammatory therapy. Majority pharmacological studies have supported their medicinal use of Annonaceae family in traditional medicine against pain (Badrie and Schauss, 2010; Cercato et al., 2015), anthelmintic (Auddy et al., 2003; Bhalke and Chavan, 2011), malaria (Duke, 2000; Garavito et al., 2006), and weight loss (Cercato et al., 2015). Further studies should be carried out to elucidate the exact composition of plant extracts to standardize the formulations based on ingredients. The randomized human trial should be conducted with compounds with superior IC50 to convert their pre-clinical results into clinical data. This will help us to develop a better understanding of the pharmacokinetics and dynamics, bioavailability, and toxicity associated with their use in clinical setting.

## Author contributions

All authors listed have made a substantial, direct and intellectual contribution to the work, and approved it for publication.

### Conflict of interest statement

The authors declare that the research was conducted in the absence of any commercial or financial relationships that could be construed as a potential conflict of interest.
